# Theory based interventions for caries related sugar intake in adults: systematic review

**DOI:** 10.1186/s40359-017-0194-z

**Published:** 2017-07-25

**Authors:** Said Hartih Al Rawahi, Koula Asimakopoulou, Jonathon Timothy Newton

**Affiliations:** Social & Behavioural Sciences Unit, Department of Population and Patient Health, Dental Institute, King’s College London, Tower Wing, London, SE1 9RT UK

**Keywords:** Social cognition model, Behavioural science, Adult, Dental caries, Free sugar intake, Systematic review

## Abstract

**Background:**

Theories of behavior change are essential in the design of effective behaviour change strategies. No studies have assessed the effectiveness of interventions based on psychological theories to reduce sugar intake related to dental caries. The study assessed the effect of interventions based on Social Congition Models (SCMs) on sugar intake in adults, when compared with educational interventions or no intervention.

**Methods:**

A range of papers were considered: Systematic review Systematic Reviews with or without Meta Analyses; Randomised Controlled Trials; Controlled Clinical Trials and Before and after studies, of interventions based on Social Cognition Models aimed at dietary intake of sugar in adults. The Cochrane database including: Oral Health Group’s Trials Register (2015), MEDLINE (from 1966 to September 2015), EMBASE (from 1980 to September 2015), PsycINFO (from 1966 to September 2015) were searched.

**Results:**

No article met the full eligibility criteria for the current systematic review so no articles were included.

**Conclusion:**

There is a need for more clinical trials to assess the effectiveness of interventions based on psychological theory in reducing dietary sugar intake among adults.

**Systematic Review Protocol Registration:**

PROSPERO: CRD42015026357.

**Electronic supplementary material:**

The online version of this article (doi:10.1186/s40359-017-0194-z) contains supplementary material, which is available to authorized users.

## Background

Theories of behavior change [1–4] are essential in the design of effective behaviour change strategies. Such theories [5, 6] can be helpful in improving our understanding of how behaviour change might lead to a healthy lifestyle. Interventions based on such models have been shown to predict behaviour change better than non-theory based interventions [7].

Social Cognition Models (SCMs) are a subgroup of psychological theories, which are based on the assumption that the individuals’ attitudes and beliefs towards a behaviour are strongly predictive of the likelihood of them engaging in that behaviour [8]. Interventions based on such models have been shown to improve dietary behaviours related to general health in highly selected patient groups. For example, Stacey and his colleagues [9] conducted a systematic review and meta-analysis to assess the effectiveness of physical activity and dietary change interventions based on Social Cognitive Theory among individuals who had survived a cancer diagnosis. The study showed that most of the included interventions were effective for enhancing dietary behaviour and physical activity. The authors, concluded that interventions based on psychological theories are effective in changing behaviour.

In oral health, two comprehensive systematic reviews have been conducted to assess the effectiveness of interventions based on SCMs, which aimed to improve adherence to oral hygiene related behaviours in adults with periodontal diseases. In the first systematic review, Renz and colleagues [10] reported that the low quality of studies associated with SCTs, made it difficult to draw any conclusions about SCT model efficacy. In the second systematic review, Newton and Asimakopoulou [11] identified that self-efficacy, goal setting, and planning were the most effective constructs for improving oral health behaviour in periodontal patients. This suggests that at least some components of SCMs may be effective for predicting oral health behaviors regardless of the overall theoretical framework which they were part of [12].

However, upto date there is no published systematic review of the effectiveness of interventions based on psychological models of health related behaviour to reduce sugar intake related to dental caries in adults. Dental caries is a prevalent issue that affects the majority of the adult population around the world [13–15]; for instance in the US more than 84% of adults have some caries experience [16] and the average Decayed, Missing, Filling Tooth (DMFT) score of adults in the UK of adults aged between 35 and 44 year olds is 11.57 [17, 18]. On the basis of a systematic review, Moynihan and Kelly [19] concluded that reducing daily free sugars intake to less than 10% of total energy would reduce the prevalence of dental caries; a further reduction to less than 5% may prevent the progression of dental caries in the long-term. The relationship between sugar intake and caries remains strong even with the application of fluoride as a preventive strategy [19], emphasizing the importance of lifestyle interventions to reduce sugar intake.

Achieving the target consumption of free sugars is likely to require behaviour change by individuals, and the dental team can play an important part in assisting people to achieve this. The aim of the current systematic review is to examine the effectiveness of interventions based on Social Congitive Models (SCMs), aimed at reducing sugar intake related to dental caries among adults. The review aims to rectify this by addressing the following question: What is the effect of interventions based on Social Congitive Models (SCMs) on sugar intake in adults, when compared with educational interventions or no intervention?

## Methods

The current systematic review was registered with the International Prospective Register of Systematic Reviews (PROSPERO), 2015 database (CRD42015026357). The reporting of the review is based on the Preferred Reporting Items for Systematic Review and Meta-Analysis (PRISMA) [20].

Eligibility criteria:

- *Types of studies*
  - ○ Systematic Reviews with or without Meta Analysis
  - ○ Randomised Controlled Trials
  - ○ Controlled Clinical Trials
  - ○ Before and after studies
- *Types of interventions*

This review included interventions based on the following psychological theories and models of health related behaviour:

- ○ Health Belief Model (HBM)
- ○ Theory of Reasoned Action (TRA)
- ○ Theory of Planned Behaviour (TPB)
- ○ Self Efficacy Model
- ○ Transtheoretical Model (Stages of Change)
- ○ Protection Motivation Model
- ○ Health Locus of Control (HLOC)
- ○ Implementation Intentions
- ○ PRIME (Plans, Responses, Impulses, Motives, Evaluation) Theory of Motivation
- ○ Unrealistic Optimism Bias
- ○ Self Regulatory Model
- ○ Health Action Process Approach (HAPA)
- ○ Precaution Adoption Process Model (PAPM)
- ○ Outcome Expectancy
- ○ Hypothesis Model of Compliance
- ○ Social Cognitive Theory
- ○ Information Motivation Behaviour Skills Model (IMBM)
- ○ Operant and Classical Conditioning
- ○ Interventions adopting techniques from Cognitive Behaviour Therapy
- ○ Motivational Interviewing
- ○ COM-B (Capabilities, Opportunities, Motivations, Behaviour) Model
- ○ Behaviour Change Wheel (BCW)
• Papers were included if they clearly stated that one of the above psychological models or theories was used and at least one construct identified in the theory or the model was targeted in the intervention.
• Sugars were defined “as any of: total sugars, free sugars, added sugars, sucrose, non-milk extrinsic (NME) sugars, expressed as g or kg/day or /yr or as percentage E.” [19; p.1]
• *Comparison:* oral health educational (non-psychological theory based) interventions, or no intervention controls.
• *Types of participants*
  - Adults aged 18 or over.
  - Patients with or without dental caries. For the aim of this review, dental caries is defined on the basis of diagnosis from a dental clinician. This includes diagnoses of any caries lesion active, progressive or arrested, which includes root caries.
- Outcome measures:
  Three outcome measures were considered to determine adults oral health related behaviours for this review [21].
- *Behavioural outcomes*: reduction of sugar intake, assessed by any method, including self-report, food diary, observation etc.
- *Attitude and belief outcomes:*
- *Primary outcomes:* Patients’ attitudes, beliefs and their intentions towards sugar intake related to dental caries.
- *Clinical status outcomes:* Progression of dental caries in the permanent dentition, assessed via tooth decay increment: DMFS (Decayed, Missing, Filling, Surface) and/or DMFT scores; filled teeth including replaced restorations; early carious lesions which are arrested or reversed; root caries.

Information sources

- The Cochrane database including: Oral Health Group’s Trials Register (2015),
- MEDLINE (from 1966 to September 2015),
- EMBASE (from 1980 to September 2015),
- PsycINFO (from 1966 to September 2015).

The search included reference lists from relevant articles and the eligible authors of trials were contacted for additional information if necessary. The search was not restricted to a particular language.

### Search

A detailed search strategy was developed from Medline. An information specialist was consulted to assist with the development of the search strategy, as previous research suggests this improves the quality of the search [22]. This search strategy was amended accordingly for use on each of the other selected databases. MeSH (fixed vocabulary) and free text terms will be used to conduct the search strategy. Additional file 1 lists the search terms, which were adopted.

### Study selection

Two authors (S Al and JTN) conducted the search and assessed the studies, initially through evaluating titles, keywords, and abstracts. Any articles, which were not considered to be suitable, were rejected at this stage. Full reports of studies were retrieved for all studies if they met the inclusion criteria. Further full review was conducted if the studies met the inclusion criteria for full assessment.

### Data collection process

Data were collected for each study on a data sheet, which includes the following data points:

- Study Design
- Sample size
- Psychological constructs assessed and theoretical framework adopted
- Measures of primary and secondary outcomes
- Effect of intervention on outcomes

Two authors (JTN and SAL independently extracted the data, following the guidance of the Cochrane reviewers’ handbook checklist [22].

### Risk of bias in individual studies

The Cochrane reviewers’ handbook checklist was to be used [23] to assess the risk of bias interventional trials.

### Synthesis of data

A meta-analysis was planned if a sufficient number of homogeneous studies met the inclusion criteria.

## Results

### Description of studies

**Fig. 1:**
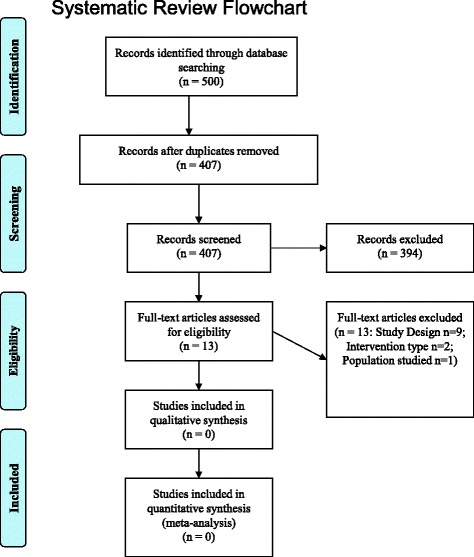
Systematic review flowchart

Initially, the search strategy identified 500 articles (see Fig. 1- Systematic Review Flowchart). After exclusion of duplicates, the titles and abstracts of 407 articles were screened for relevance. At this stage 13 papers were apparently relevant being related to dentistry and having applied psychological models and theories to develop the reported intervention. However, after obtaining the full manuscripts no article met the full eligibility criteria for the current systematic review. Table 1 provides the characteristics of the excluded studies.

**Table 1:** Characteristics of excluded studies

Reference	Paper Title	Participants	Study Design	Psychological Model	Reasons for exclusion
Reisine et al. (1994) [27]	A biopsychosocial model to predict caries in preschool children	Children & parents	Cross-sectional survey	None specified	Cross-sectional study
Astrøm & Rise (1996) [28]	Analysis of adolescents’ beliefs about the outcome of using dental floss and drinking non-sugared mineral water.	Adolescents	Cross-sectional survey	None specified	Cross-sectional study and participants were adolescents
Astrøm, Awadia & Bjorvatn (1999) [29]	Perceptions of susceptibility to oral health hazards: a study of women in different cultures.	Adults	Cross-sectional survey	None specified	Cross-sectional study
Roberts, Blinkhorn & Duxbury (2003) [30]	The power of children over adults when obtaining sweet snacks.	Children & parents	Cross-sectional survey	Theory of Reasoned Action	Cross-sectional study
Adair et al. (2004) [31]	Familial and cultural perceptions and beliefs of oral hygiene and dietary practices among ethnically and socio-economically diverse groups.	Children	Cross-sectional survey	Theory of Planned Behaviour, Health Belief Model andthe Health Locus of Control	Cross-sectional study and participants were children
Astrom (2004) [32]	Validity of Cognitive Predictors of Adolescent Sugar Snack Consumption.	Adolescents	Cross-sectional survey	Theory of planned behaviour	Cross-sectional study and participants were adolescents.
Astrøm AN, & Okullo I., (2004) [33]	Temporal stability of the theory of planned behavior: a prospective analysis of sugar consumption among Ugandan adolescents.	Adolescents	Cross-sectional survey	Theory of planned behaviour	Cross-sectional study
Skeie et al., (2006) [34]	Parental risk attitudes and caries-related behaviours among immigrant and western native children in Oslo.	Children & parents	Cross-sectional survey	Theory of planned behaviour, Sociallearning theory and the Health Belief Model. Health Locus of Control	Cross-sectional study
Astrøm & Kiwanuka (2006) [35]	Examining intention to control preschool children’s sugar snacking: a study of carers in Uganda.	Children	Cross-sectional survey	Theory of planned behaviour	Cross-sectional study and participants were children
Vanagas et al. (2009) [36]	Associations between parental skills and their attitudes toward importance to develop good oral hygiene skills in their children.	Adults	Cross-sectional survey	Theory of Planned Behaviour, Health Belief Model and the Health Locus of Control model,	Cross-sectional study
Tolvanen et al. (2009) [37]	Changes in children’s oral health-related behavior, knowledge and attitudes during a 3.4-yr. randomized clinical trial and oral health-promotion program.	Children	RCT	None specified	Participants were children and no Social Cognition Models identified
Harris et al. (2012) [24]	One-to-one dietary interventions undertaken in a dental setting to change dietary behaviour.	All ages	Systematic Review (S.R)	None specified	No Social Cognition Models identified
Weber-Gasparoni et al. (2013) [38]	An effective psychoeducational intervention for early childhood caries prevention: part 1	Children & parents	RCT	Self-determination theory (SDT)	Participants were children
Weber-Gasparoni et al. (2013) [39]	An effective psychoeducational intervention for early childhood caries prevention: part 2	Children & parents	RCT	Self-determination theory (SDT)	Participants were children

### Risk of bias and data synthesis

Given that there were no papers meeting the criteria for the review, risk of bias and synthesis of data were not conducted.

## Discussion

This review sought to assess the effectiveness of interventions based on social cognition models (SCMs) to reduce sugar consumption among adults. The review focused on an often neglected area of health psychology that of oral health. No studies were found that matched the inclusion criteria of the review.

There is a dearth of intervention studies designed to explore the effectiveness of psychologically based interventions on oral health including oral hygiene as well as diet related behaviour. Harris and his colleagues [24] examined the effectiveness of one-to-one dietary interventions for dietary behavior among all age groups in dental settings. They identified five studies, none of which included the modification of constructs identified from psychological models of behaviour. Similarly Renz et al. [10], Werner et al. [25] and Newton and Asimakopoulou [11] located very few trials of interventions to enhance oral health related behaviours (toothbrushing and flossing) based on psychological theory, echoing calls for more and better-designed trials [26].

Whilst it is disappointing that no intervention studies based on psychological theoretical models were identified from our systematic search, the current review has confirmed the need for high quality, theory-driven interventions to support clinical practice and has highlighted potential opportunities for researchers and intervention designers to explore and examine such approaches.

## Conclusion

To date there has been no published study of the effectiveness of interventions based on Social Cognition Models (SCMs) aimed at reducing sugar intake related to dental caries among adults. Given the contribution of dietary sugars to caries development and the role of lifestyle change to combat dietary sugar intake, there is a need for trials of theory-based interventions aimed at reducing individuals’ consumption of dietary sugars.

## Additional file

Additional file 1: Keywords search strategy. (DOCX 123 kb)
