# Supplementary material for: Theory based interventions for caries related sugar intake in adults: systematic review
Source: BMC Psychol. 2017 Jul 25;5:25. doi: 10.1186/s40359-017-0194-z (PMC5526314; doi:10.1186/s40359-017-0194-z)
Supplement: Additional file 1: — Keywords search strategy. (DOCX 123 kb) [file 40359_2017_194_MOESM1_ESM.docx]

**Keywords Search Strategy:**

**A) Suggested Search Terms for Medline; PsycINFO; and Embase Classic+Embase :**

1. Psychol$.mp. [mp=ti, ab, hw, tc, id, ot, tm, tn, dm, mf, dv, kw, nm, kf, px, rx, ui]
2. Cognit$.mp. [mp=ti, ab, hw, tc, id, ot, tm, tn, dm, mf, dv, kw, nm, kf, px, rx, ui]
3. Health belief$.mp. [mp=ti, ab, hw, tc, id, ot, tm, tn, dm, mf, dv, kw, nm, kf, px, rx, ui]
4. Theory of planned behaviour.mp. [mp=ti, ab, hw, tc, id, ot, tm, tn, dm, mf, dv, kw, nm, kf, px, rx, ui]
5. Theory of reasoned action.mp. [mp=ti, ab, hw, tc, id, ot, tm, tn, dm, mf, dv, kw, nm, kf, px, rx, ui]
6. Self efficacy.mp. [mp=ti, ab, hw, tc, id, ot, tm, tn, dm, mf, dv, kw, nm, kf, px, rx, ui]
7. Transtheoretical model.mp. [mp=ti, ab, hw, tc, id, ot, tm, tn, dm, mf, dv, kw, nm, kf, px, rx, ui]
8. Stages of change.mp. [mp=ti, ab, hw, tc, id, ot, tm, tn, dm, mf, dv, kw, nm, kf, px, rx, ui]
9. Locus of control.mp. [mp=ti, ab, hw, tc, id, ot, tm, tn, dm, mf, dv, kw, nm, kf, px, rx, ui]
10. Internal‐External Control.mp. [mp=ti, ab, hw, tc, id, ot, tm, tn, dm, mf, dv, kw, nm, kf, px, rx, ui]
11. Self‐regulatory model.mp. [mp=ti, ab, hw, tc, id, ot, tm, tn, dm, mf, dv, kw, nm, kf, px, rx, ui]
12. Implementation intentions.mp. [mp=ti, ab, hw, tc, id, ot, tm, tn, dm, mf, dv, kw, nm, kf, px, rx, ui]
13. Protection motivation.mp. [mp=ti, ab, hw, tc, id, ot, tm, tn, dm, mf, dv, kw, nm, kf, px, rx, ui]
14. Optimistic bias.mp. [mp=ti, ab, hw, tc, id, ot, tm, tn, dm, mf, dv, kw, nm, kf, px, rx, ui]
15. Unrealistic optimism.mp. [mp=ti, ab, hw, tc, id, ot, tm, tn, dm, mf, dv, kw, nm, kf, px, rx, ui]
16. Health action process approach.mp. [mp=ti, ab, hw, tc, id, ot, tm, tn, dm, mf, dv, kw, nm, kf, px, rx, ui]
17. Information Motivation Behaviour$.mp. [mp=ti, ab, hw, tc, id, ot, tm, tn, dm, mf, dv, kw, nm, kf, px, rx, ui]
18. Information‐Motivation‐Behaviour$.mp. [mp=ti, ab, hw, tc, id, ot, tm, tn, dm, mf, dv, kw, nm, kf, px, rx, ui]
19. Precaution adoption process model.mp. [mp=ti, ab, hw, tc, id, ot, tm, tn, dm, mf, dv, kw, nm, kf, px, rx, ui]
20. Social Cognitive Theory.mp. [mp=ti, ab, hw, tc, id, ot, tm, tn, dm, mf, dv, kw, nm, kf, px, rx, ui]
21. "Conditioning (psychology)".mp. [mp=ti, ab, hw, tc, id, ot, tm, tn, dm, mf, dv, kw, nm, kf, px, rx, ui]
22. Social learning Theory.mp. [mp=ti, ab, hw, tc, id, ot, tm, tn, dm, mf, dv, kw, nm, kf, px, rx, ui]
23. conditioning classical.mp. [mp=ti, ab, hw, tc, id, ot, tm, tn, dm, mf, dv, kw, nm, kf, px, rx, ui]
24. ￼Conditioning operant.mp. [mp=ti, ab, hw, tc, id, ot, tm, tn, dm, mf, dv, kw, nm, kf, px, rx, ui]
25. (behavio$ adj 4 intention$).mp. [mp=ti, ab, hw, tc, id, ot, tm, tn, dm, mf, dv, kw, nm, kf, px, rx, ui]
26. (behavio$ adj4 modificat$).mp. [mp=ti, ab, hw, tc, id, ot, tm, tn, dm, mf, dv, kw, nm, kf, px, rx, ui]
27. (behavio$ adj4 change$).mp. [mp=ti, ab, hw, tc, id, ot, tm, tn, dm, mf, dv, kw, nm, kf, px, rx, ui]
28. BEHAVIOR THERAPY.mp. [mp=ti, ab, hw, tc, id, ot, tm, tn, dm, mf, dv, kw, nm, kf, px, rx, ui]
29. COGNITIVE THERAPY.mp. [mp=ti, ab, hw, tc, id, ot, tm, tn, dm, mf, dv, kw, nm, kf, px, rx, ui]
30. Patient education.mp. [mp=ti, ab, hw, tc, id, ot, tm, tn, dm, mf, dv, kw, nm, kf, px, rx, ui]
31. PRIME Theory.mp. [mp=ti, ab, hw, tc, id, ot, tm, tn, dm, mf, dv, kw, nm, kf, px, rx, ui]
32. ￼PRIME Theory of Motivation.mp. [mp=ti, ab, hw, tc, id, ot, tm, tn, dm, mf, dv, kw, nm, kf, px, rx, ui]
33. COM‐B Model.mp. [mp=ti, ab, hw, tc, id, ot, tm, tn, dm, mf, dv, kw, nm, kf, px, rx, ui]
34. COM‐B System.mp. [mp=ti, ab, hw, tc, id, ot, tm, tn, dm, mf, dv, kw, nm, kf, px, rx, ui]
35. Behaviour Change Wheel.mp. [mp=ti, ab, hw, tc, id, ot, tm, tn, dm, mf, dv, kw, nm, kf, px, rx, ui]
36. 1 or 2 or 3 or 4 or 5 or 6 or 7 or 8 or 9 or 10 or 11 or 12 or 13 or 14 or 15 or 16 or 17 or 18 or 19 or 20 or 21 or 22 or 23 or 24 or 25 or 26 or 27 or 28 or 29 or 30 or 31 or 32 or 33 or 34 or 35
37. Dental caries.mp. [mp=ti, ab, hw, tc, id, ot, tm, tn, dm, mf, dv, kw, nm, kf, px, rx, ui]
38. caries.mp. [mp=ti, ab, hw, tc, id, ot, tm, tn, dm, mf, dv, kw, nm, kf, px, rx, ui]
39. tooth decay.mp. [mp=ti, ab, hw, tc, id, ot, tm, tn, dm, mf, dv, kw, nm, kf, px, rx, ui]
40. caries.ab. or caries.ax. or caries.bt. or caries.ce. or caries.cf. or caries.cm. or caries.cq. or caries.cs. or caries.ct. or caries.cu. or caries.cv. or caries.dv. or caries.id. or caries.in. or caries.ja. or caries.ji. or caries.jw. or caries.jx. or caries.kf. or caries.kw. or caries.nm. or caries.nw. or caries.ot. or caries.tc. or caries.ti. or caries.tm.
41. exp tooth decay/
42. ￼tooth decay$.mp. [mp=ti, ab, hw, tc, id, ot, tm, tn, dm, mf, dv, kw, nm, kf, px, rx, ui]
43. exp DMF/
44. 37 or 38 or 39 or 40 or 41 or 42 or 43
45. exp dietary carbohydrate/
46. carbohydrat$.ab,ti.
47. sugar$.ab,ti.
48. ((Glucose or fructose or lactose or maltose or sucrose) adj3 (diet$ or intake$)).tw.
49. sucrose/
50. exp starch/
51. Starch$.tw.
52. polysaccharide$.tw.
53. monosaccharide$.tw.
54. disaccharide$.tw.
55. oligosaccharide$.tw.
56. polysaccharides/
57. inulin$.tw.
58. inulin/
59. alginates/
60. cellulose/
61. carageenan/
62. lignin/
63. methylcellulose/
64. carboxymethylcellulose/
65. maltose/
66. mannans/
67. exp oligosaccharides/
68. pectins/
69. plant gums/
70. gum arabic/
71. karaya gum/
72. tragacanth/
73. chitin/
74. "beta glucan$".tw.
75. beta‐glucans/
76. candy/
77. ((cake$ or biscuit$ or cookie$ or confectionery) adj3 (diet or intake)).tw.
78. carbonated beverages/
79. ((soda or carbonated or sweet$ or sugar$) adj3 beverage$).tw.
80. ((soda or carbonated or sweet$ or sugar$) adj3 drink$).tw.
81. "soft drink".tw.
82. 45 or 46 or 47 or 48 or 49 or 50 or 51 or 52 or 53 or 54 or 55 or 56 or 57 or 58 or 59 or 60 or 61 or 62 or 63 or 64 or 65 or 66 or 67 or 68 or 69 or 70 or 71 or 72 or 73 or 74 or 75 or 76 or 77 or 78 or 79 or 80 or 81
83. 36 and 44 and 82

**B) Suggested Search Terms for Cochrane database:**

1. Psychological models or psychological Theories
2. Dental caries or tooth decay or root Caries
3. Dietary carbohydrate or dietary sugar or carbohydrate food
4. 1 And 2 AND 3
5. Behavior change models or behavior Theories
6. Dental caries or tooth decay or root Caries
7. Dietary carbohydrate or dietary sugar or carbohydrate food
8. 5 And 6 AND 7
9. Psychological Theories
10. Dental caries or tooth decay or root Caries or DMFT or DMF
11. Dietary carbohydrate or dietary sugar or carbohydrate food or sugary food
12. 9And 10 AND 11
13. Psychological Theories
14. Dental caries or tooth decay or root Caries
15. Dietary carbohydrate or dietary sugar
16. 13 And 14 AND 15
17. Psychological Theories
18. Dental caries or tooth decay
19. Dietary sugar
20. 17 And 18 AND 19
21. Psychological Theories
22. Dental caries or tooth decay
23. Adult* or mature or elder or old or geriatric*
24. 21 And 22 AND 23
